# Supplementary material for: LTA4H extensively associates with mRNAs and lncRNAs indicative of its novel regulatory targets
Source: PeerJ. 2023 Mar 10;11:e14875. doi: 10.7717/peerj.14875 (PMC10010175; doi:10.7717/peerj.14875)
Supplement: File S2 [file peerj-11-14875-s005.zip › Raw data files/Quality_control/FastqcView/left_frame.html]

fastqc报告


- LTA4H\_IP\_1\_fastqc
- LTA4H\_IP\_2\_fastqc
- LTA4H\_input\_1\_fastqc
- LTA4H\_input\_2\_fastqc
